# Association of dynamic platelet-to-lymphocyte ratio trajectories using group-based trajectory modeling with short-term outcomes and survival in patients undergoing laparoscopic radical resection for rectal cancer: a retrospective study

**DOI:** 10.3389/fonc.2026.1819803

**Published:** 2026-07-27

**Authors:** Bao Lin, Shijie Sun, Hongjian Zhang, Changqing Lin, Yuanlong Chi

**Affiliations:** Department of Gastrointestinal Surgery, Sanming First Hospital Affiliated to Fujian Medical University, Sanming, Fujian, China

**Keywords:** dynamic changes, group-based trajectory modeling, laparoscopic radical resection, platelet-to-lymphocyte ratio, prognosis, rectal cancer

## Abstract

**Purpose:**

To investigate the association of dynamic platelet-to-lymphocyte ratio (PLR) change trajectories, grouped via Group-Based Trajectory Modeling (GBTM), with short-term outcomes and survival prognosis in patients undergoing laparoscopic radical resection for rectal cancer.

**Methods:**

Clinical data of patients who underwent laparoscopic radical resection for rectal cancer at Sanming First Hospital Affiliated to Fujian Medical University between February 2016 and January 2020 were retrospectively analyzed. GBTM was applied to PLR values measured at five time points: preoperatively (T0) to before the fourth postoperative adjuvant chemotherapy session (T4), to identify distinct PLR trajectories. Clinicopathological characteristics, perioperative indicators, complications, and survival were compared among trajectory groups.

**Results:**

GBTM identified three distinct PLR trajectories: Increasing Group (IG, n=67, 27.2%), Increasing-Decreasing Group (IDG, n=90, 36.6%), and Decreasing Group (DG, n=89, 36.2%). The incidence of grade II or higher postoperative complications was significantly lower in the DG group (7.9%) compared to the IG (20.9%) and IDG groups (11.1%) (P = 0.046). The 5-year overall survival (OS) rate for the entire cohort was 68.3%. Survival analysis showed that the 5-year OS rates of patients in the DG and IDG groups were significantly higher than that in the IG group (P < 0.05).Multivariate Cox analysis identified age ≥65 years (HR = 1.803, P = 0.017), N2 stage (HR = 1.724, P = 0.041), and poor differentiation (HR = 2.009, P = 0.003) as independent risk factors. Compared to the IG group, the IDG (HR = 0.497, P = 0.012) and DG groups (HR = 0.365, P = 0.001) remained protective factors in the multivariable-adjusted Cox model.

**Conclusion:**

PLR exhibits distinct dynamic change trajectories from surgery to chemotherapy in rectal cancer patients. The persistently decreasing PLR pattern identified by GBTM is associated with lower complication rates and superior long-term survival, suggesting its potential utility in postoperative risk stratification.

## 1 Introduction

Colorectal cancer (CRC) is the third most common malignant tumor globally, with rectal cancer accounting for approximately 30% to 40% of CRC cases (1). In recent years, laparoscopic radical resection for rectal cancer has become the standard surgical approach due to its advantages of minimal invasiveness and faster recovery (2). While the widespread adoption of laparoscopic techniques and the optimization of comprehensive treatment strategies have significantly increased the rate of radical resection and perioperative safety in rectal cancer patients, marked variation persists in postoperative recurrence, metastasis, and prognosis (3, 4). Therefore, identifying reliable biomarkers for more accurate prediction of short-term outcomes and long-term prognosis is crucial for formulating individualized treatment decisions. Research indicates that systemic inflammatory response plays a significant role in tumor microenvironment regulation, immune escape, and metastasis. Inflammatory markers, represented by the platelet-to-lymphocyte ratio (PLR), have been demonstrated to correlate with the prognosis of various malignancies (5–8).

However, existing studies predominantly assess tumor prognosis based on PLR measured at a single time point. Surgery and postoperative adjuvant therapy can induce dynamic changes in PLR, rendering measurements from a single time point insufficient to comprehensively reflect the dynamic changes in the body’s inflammatory state (9). Group-Based Trajectory Modeling (GBTM) provides an innovative method for analyzing the dynamic evolution of biomarkers by identifying subgroups of individuals with similar longitudinal change patterns (10). This method has been used in cancer-related studies (11, 12). For example, Feng et al. (13) found in a Chinese population without diabetes that different fasting blood glucose trajectories were significantly associated with cancer risk; compared with the moderate-stable group, the high-stable group had a 66% increased risk of gastrointestinal cancer. In addition, recent studies have shown that dynamic changes in inflammatory markers can more accurately predict tumor treatment outcomes than static measurements. For instance, the trajectory of the neutrophil-to-lymphocyte ratio (NLR) during neoadjuvant chemotherapy was confirmed to be significantly associated with survival in patients with gastric cancer (14). Currently, the dynamic trajectory of PLR in rectal cancer patients from surgery to adjuvant chemotherapy remains unclear, and the relationship between PLR trajectories and surgical complications, tumor recurrence, and survival outcomes requires further investigation. This study applies GBTM to model the dynamic trajectories of PLR during the perioperative period and adjuvant chemotherapy in rectal cancer patients. It aims to reveal the clinical characteristics associated with distinct PLR trajectories and explore their association with short-term surgical outcomes and long-term survival outcomes.

## 2 Materials and methods

### 2.1 Study subjects

A retrospective analysis was performed on the clinical data of rectal cancer patients treated at the Sanming First Hospital Affiliated to Fujian Medical University between February 2016 and January 2020. Patient inclusion criteria were: (1) age between 45 and 80 years. (2) pathologically confirmed rectal adenocarcinoma. (3) underwent laparoscopic-assisted radical resection for rectal cancer. (4) received standard adjuvant chemotherapy postoperatively. (5) postoperative survival time>6 months. (6) availability of complete clinical and follow-up data. Exclusion criteria were: (1) severe cardiac or pulmonary insufficiency or unstable comorbidities. (2) active infectious diseases, autoimmune diseases, hematological disorders, or other conditions potentially significantly affecting inflammatory markers. (3) receipt of neoadjuvant chemoradiotherapy. (4) presence of distant metastases (e.g., liver, lung) at diagnosis. (5) history of or concurrent other malignancies. This study received approval from the Ethics Committee of the Sanming First Hospital Affiliated to Fujian Medical University (No. IRB-2023-48). Written informed consent was waived due to the retrospective nature of the study.

### 2.2 Data collection

A standardized data collection form was designed. Two attending gastrointestinal surgeons independently extracted clinical data from the hospital’s electronic medical record system. The collected data underwent cross-checking, and any discrepancies were adjudicated by a third senior surgeon. The collected data included sex, age, American Society of Anesthesiologists (ASA) physical status classification, Body Mass Index (BMI), comorbidities, surgical approach, tumor size, tumor stage based on postoperative pathological findings according to the 8th edition of the American Joint Committee on Cancer (AJCC) TNM staging system (15), perineural invasion, vascular invasion, tumor differentiation grade, intraoperative blood loss, operative time, postoperative hospital stay, total retrieved lymph nodes, and postoperative complications occurring within 30 days (classified according to the Clavien-Dindo (CD) grading system (16).

### 2.3 Adjuvant chemotherapy and inflammatory marker measurement

All enrolled patients received adjuvant chemotherapy with the CAPOX regimen: oxaliplatin 130 mg/m² intravenously on day 1, plus capecitabine 1000 mg/m² orally twice daily on days 1-14, repeated every 21 days per cycle. Fasting peripheral venous blood samples were collected at five time points: preoperatively (T0), before the first (T1), second (T2), third (T3), and fourth (T4) cycles of adjuvant chemotherapy. Complete blood counts were analyzed using an automated hematology analyzer. Platelet count (×10^9^/L) and absolute lymphocyte count (×10^9^/L) were recorded. The platelet-to-lymphocyte ratio (PLR) was calculated as: PLR = platelet count/absolute lymphocyte count.

### 2.4 Follow-up

Patients were followed up via telephone, WeChat communication, outpatient visits, and inpatient medical records. The follow-up schedule was: every 3–6 months for the first 3 years postoperatively, then annually thereafter. Follow-up assessments included physical examination, complete blood count, blood biochemistry, carcinoembryonic antigen (CEA), carbohydrate antigen 19-9 (CA19-9), contrast-enhanced CT or MRI of the chest/abdomen/pelvis, and annual colonoscopy. Survival status and date of death (if applicable) were recorded. Follow-up concluded on January 31, 2025. Overall survival (OS) was defined as the time from surgery to death from any cause or the last follow-up date. Seven patients were lost to follow-up, yielding a loss-to-follow-up rate of 2.8%.

### 2.5 Statistical analysis

Continuous variables with normal distribution are presented as mean ± standard deviation (SD) and compared using independent samples t-test or one-way ANOVA. Non-normally distributed variables are expressed as median and interquartile range [Median (IQR)] and compared using Mann-Whitney U test or Kruskal-Wallis H test. Categorical variables are presented as frequency (%) and compared using Pearson’s χ² test or Fisher’s exact test. The optimal PLR cutoff value was determined by receiver operating characteristic (ROC) curve analysis, maximizing the Youden index. GBTM was applied to analyze the dynamic changes in PLR across five time points (T0 to T4). Modeling was performed using the traj package in Stata (version 15.0; StataCorp LLC, College Station, TX, USA). A baseline model without covariates was constructed. The optimal number of trajectory groups (2–3 groups) and polynomial order (linear, quadratic, cubic) for each group were determined by minimizing the Bayesian Information Criterion (BIC) and Akaike Information Criterion (AIC), while ensuring clinical interpretability. Model fit was assessed using: (1) average posterior probability (AvePP) > 0.7 per group, and (2) odds of correct classification (OCC) > 5.0.

Survival curves were generated using the Kaplan-Meier method, with between-group differences assessed by log-rank test. To control for confounding bias arising from the imbalanced distribution of T stage and N stage among different PLR trajectory groups, we constructed three stepwise−adjusted Cox proportional hazards regression models to evaluate the impact of trajectory groups on overall survival. Furthermore, we added interaction terms between trajectory groups and TNM stage into the models and assessed the statistical significance of the interactions using the likelihood ratio test. Statistical analyses were performed using IBM SPSS Statistics (version 27.0; IBM Corp., Armonk, NY, USA) and R software (version 4.3.3; R Foundation for Statistical Computing, Vienna, Austria). A two-sided P < 0.05 was considered statistically significant.

## 3 Results

### 3.1 GBTM grouping results and clinicopathological characteristics

**Figure 1:**
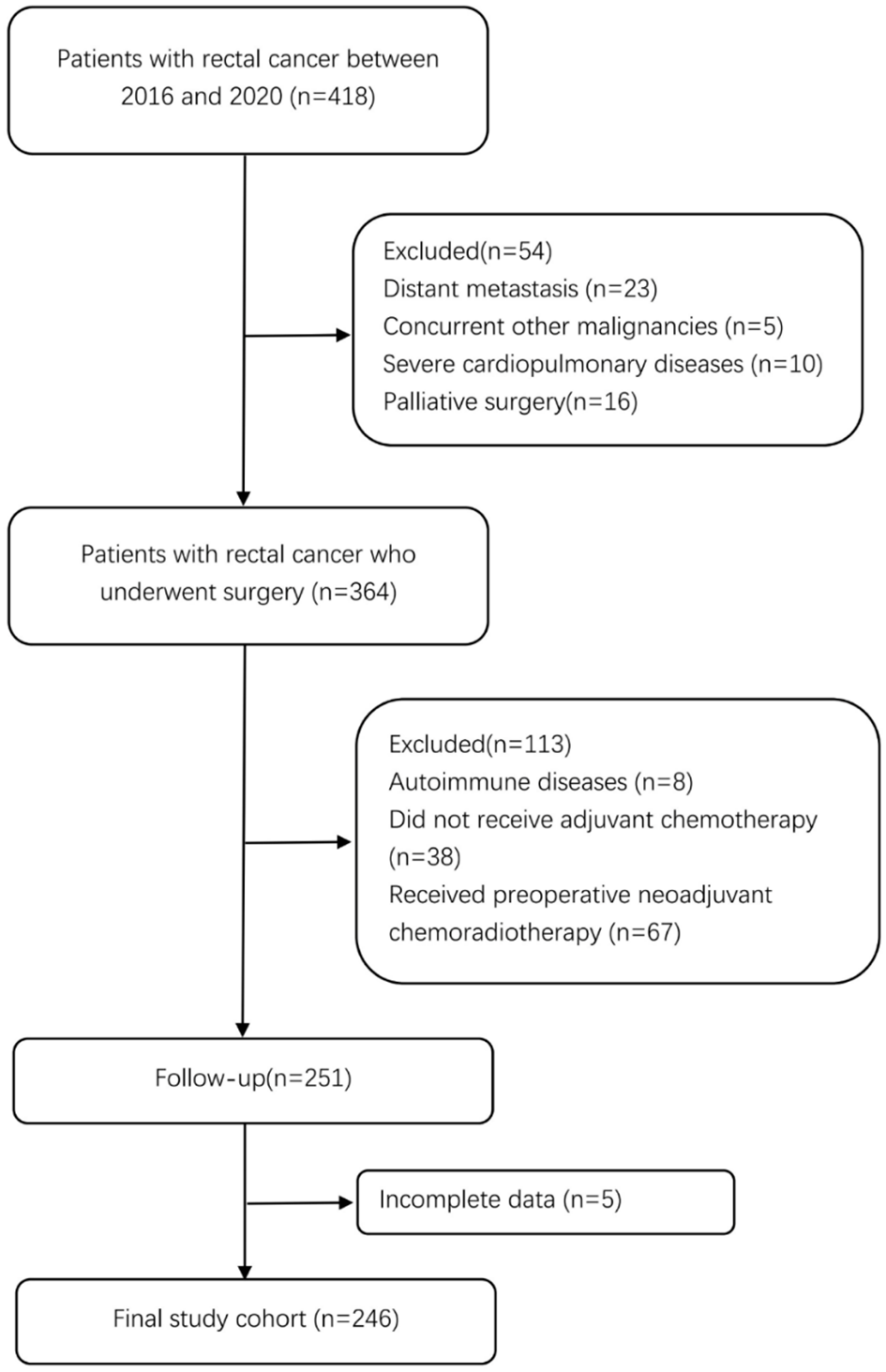
Flowchart of patient selection for the study cohort (2016–2020).

**Figure 2:**
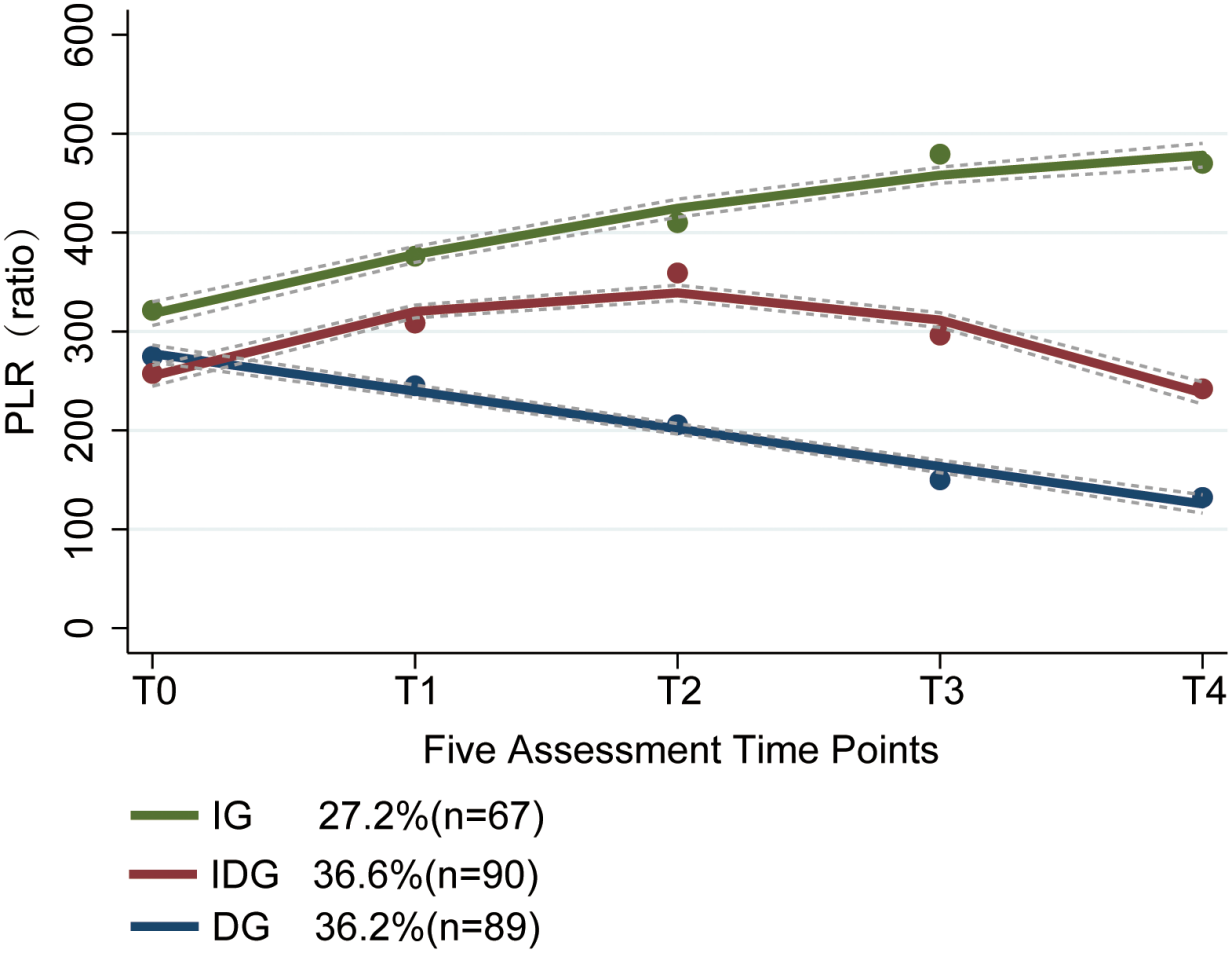
Dynamic trajectory patterns of PLR in three distinct groups.

The final cohort comprised 246 patients (**Figure 1**), including 115 males and 131 females with a mean age of 63.94 ± 11.25 years. Receiver operating characteristic analysis revealed that the PLR achieved an area under the curve (AUC) of 0.705 (95% CI: 0.637-0.772, P < 0.001) for predicting 5-year survival (**Supplementary Figure S1**). GBTM identified three distinct PLR trajectory patterns, with the three-group solution selected as optimal based on minimization of the Bayesian Information Criterion (BIC = -6874.62) (**Figure 2**). These groups were designated as: Group 1 - Increasing Group (IG, n = 67, 27.2%), characterized by progressive PLR elevation; Group 2 - Increasing-Decreasing Group (IDG, n = 90, 36.6%), showing initial rise followed by decline; and Group 3 - Decreasing Group (DG, n = 89, 36.2%), demonstrating consistent PLR reduction. All groups exhibited robust model fit parameters, with AvePP > 0.7 and OCC > 5.0 (**Supplementary Tables S1**–**S3**). Comparative analysis demonstrated no statistically significant differences (P > 0.05) among the trajectory groups regarding sex, age, ASA classification, BMI, hypertension comorbidity, diabetes comorbidity, surgical approach, tumor size, T stage, perineural invasion, vascular invasion, or tumor differentiation grade. However, significant intergroup differences emerged in N stage and overall TNM stage distribution (P < 0.05) (**Table 1**).

**Table 1:** Baseline clinical and pathological characteristics of patients.

Characteristic	Category	IG (N = 67)	IDG (N = 90)	DG (N = 89)	F/χ²	P value
Sex	Male	27 (40.3%)	44 (48.9%)	44 (49.4%)	1.54	0.462
	Female	40 (59.7%)	46 (51.1%)	45 (50.6%)		
Age(years)	<65	34 (50.8%)	42 (46.7%)	38 (42.7%)	1.00	0.606
	≥65	33 (49.3%)	48 (53.3%)	51 (57.3%)		
ASA Class	I	37 (55.2%)	43 (47.8%)	52 (58.4%)	8.50	0.075
	II	20 (29.9%)	29 (32.2%)	32 (36.0%)		
	III	10 (14.9%)	18 (20.0%)	5 (5.6%)		
BMI (kg/m²)	<23.5	33 (49.3%)	38 (42.2%)	36 (40.5%)	1.30	0.522
	≥23.5	34 (50.7%)	52 (57.8%)	53 (59.6%)		
Hypertension	No	43 (64.2%)	51 (56.7%)	41 (46.1%)	5.25	0.073
	Yes	24 (35.8%)	39 (43.3%)	48 (53.9%)		
Diabetes	No	57 (85.1%)	75 (83.3%)	73 (82.0%)	0.26	0.880
	Yes	10 (14.9%)	15 (16.7%)	16 (18.0%)		
Surgical Approach	Open	2 (3.0%)	0(0%)	3(3.4%)		0.228*
	Laparoscopic	65 (97.0%)	90 (100%)	86 (96.6%)		
Tumor Size (cm)	<3.5	38 (56.7%)	62 (68.9%)	60 (67.4%)	2.85	0.241
	≥3.5	29 (43.3%)	28 (31.1%)	29 (32.6%)		
CEA		3.65 ± 1.35	3.99 ± 1.13	3.82 ± 1.18	1.60	0.205
CA199		22.07 ± 9.55	21.59 ± 10.03	23.02 ± 8.76	0.54	0.586
T Stage	T1	1 (1.5%)	1 (1.1%)	0 (0.00%)		0.230*
	T2	1 (1.5%)	3 (3.3%)	5 (5.6%)		
	T3	32 (47.8%)	53 (58.9%)	55 (61.8%)		
	T4	33 (49.3%)	33 (36.7%)	29 (32.6%)		
N Stage	N0	26 (38.8%)	53 (58.9%)	53 (59.6%)	23.17	<0.001
	N1	10 (14.9%)	14 (15.6%)	24 (27.0%)		
	N2	31 (46.3%)	23 (25.6%)	12 (13.5%)		
TNM Stage	II	26 (38.8%)	53 (58.9%)	53 (59.6%)	8.18	0.017
	III	41 (61.2%)	37 (41.1%)	36 (40.5%)		
Perineural Invasion	No	45 (67.2%)	61 (67.8%)	67 (75.3%)	1.65	0.439
	Yes	22 (32.8%)	29 (32.2%)	22 (24.7%)		
Vascular invasion	No	52 (77.6%)	73 (81.1%)	69 (77.5%)	0.43	0.806
	Yes	15 (22.4%)	17 (18.9%)	20 (22.5%)		
Differentiation	Well-Moderate	49 (73.1%)	63 (70.0%)	66 (74.2%)	0.41	0.813
	Poor	18 (26.9%)	27 (30.0%)	23 (25.8%)		
Chemotherapy completion	No	12 (17.9%)	8 (8.9%)	13 (14.6%)	2.86	0.239
	Yes	55 (82.1%)	82 (91.1%)	76 (85.4%)		

ASA, American society of anesthesiologists; BMI, Body mass index; TNM, Tumor-Node-Metastasis.

* Fisher’s exact test.

### 3.2 Comparison of perioperative outcomes

No statistically significant differences were observed in intraoperative blood loss, operative time, postoperative hospital stay, or total retrieved lymph nodes among the three trajectory groups (P > 0.05). The incidence of Clavien-Dindo grade II or higher complications was 20.9% (14/67) in the IG group, 11.1% (10/90) in the IDG group, and 7.9% (7/89) in the DG group. The DG group exhibited significantly lower complication rates compared to both the IG and IDG groups (χ² = 6.18, P = 0.046) (**Supplementary Table S4**).

### 3.3 Survival analysis

**Figure 3:**
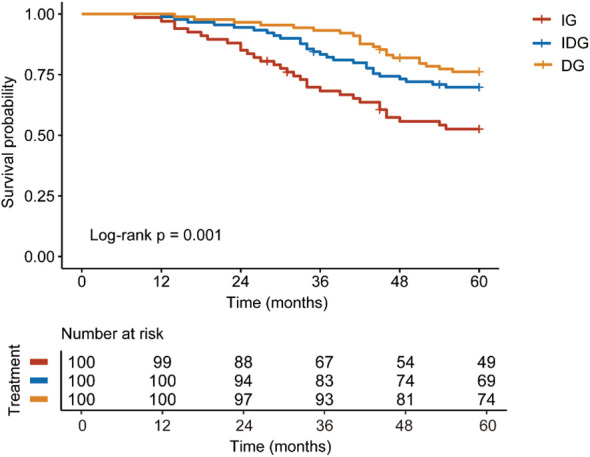
Kaplan-meier survival curves for 5-years overall survival for different trajectory groups.

Kaplan-Meier method was used to plot the survival curves for the three groups, and the log-rank test was performed for overall comparison. The results showed that the 5-year overall survival rates for the three groups were 53.7% for the IG group, 71.1% for the IDG group, and 76.4% for the DG group, with a statistically significant overall survival difference (overall log-rank P = 0.001) (**Figure 3**). Further pairwise comparisons revealed that the survival rate of the IG group was significantly lower than that of the DG group (P<0.001) and the IDG group (P = 0.013). To further explore the impact of trajectory grouping and clinical stage on prognosis, we performed subgroup analyses. The results showed that among patients with stage II rectal cancer, there was no statistically significant difference in 5-year OS among the three groups (Log-rank χ² = 1.805, P > 0.05) (**Supplementary Figure S2A**). In stage III patients, the three groups showed a statistically significant difference in 5-year OS (Log-rank χ² = 8.032, P = 0.018) (**Supplementary Figure S2B**). Further interaction analysis using a Cox regression model revealed no significant interaction between TNM stage and trajectory groups (likelihood ratio test: χ² = 3.117, P = 0.374).

### 3.4 Stepwise−adjusted Cox regression analysis for overall survival

Univariate Cox regression identified age, N stage, TNM stage, perineural invasion, vascular invasion, differentiation grade, and trajectory group as potential factors influencing 5-year OS (P < 0.05) (**Supplementary Table S5**). Given the significant differences in N stage and TNM stage among different trajectory groups, we further constructed three stepwise−adjusted Cox regression models to avoid confounding of the trajectory group effect by staging factors. The results showed that in the unadjusted Model 1, the HRs for the IDG and DG groups compared with the IG group were 0.517 (P = 0.013) and 0.391 (P < 0.001), respectively. In Model 2 (adjusted for age, T stage, N stage, perineural invasion, vascular invasion, differentiation, and chemotherapy completion), the HRs were 0.530 (P = 0.020) and 0.394 (P = 0.002), respectively. In Model 3 (with additional adjustment for CEA, CA19−9, and complication grade), the HRs were 0.497 (P = 0.012) and 0.365 (P = 0.001), respectively (**Table 2**).

**Table 2:** Cox regression analysis of trajectory groups associated with overall survival under different adjustment models.

Variable	Model 1		Model 2		Model 3	
	HR (95% CI)	P value	HR (95% CI)	P value	HR (95% CI)	P value
Trajectory group						
IG	Ref.		Ref.		Ref.	
IDG	0.517 (0.307 - 0.871)	0.013	0.530 (0.310 - 0.907)	0.020	0.497 (0.287 - 0.860)	0.012
DG	0.391 (0.225 - 0.681)	< 0.001	0.394 (0.219 - 0.709)	0.002	0.365 (0.199 - 0.671)	0.001

OR, Odds Ratio; CI, Confidence Interval.

Model 1: Unadjusted.

Model 2: Adjust: Age, T Stage, N Stage, Perineural Invasion, Vascular Invasion, Differentiation, Chemotherapy completion.

Model 3: Adjust: Age, CEA, CA199, CD grade, T Stage, N Stage, Perineural Invasion, Vascular Invasion, Differentiation, Chemotherapy completion.

## 4 Discussion

The PLR, as a novel inflammatory marker, has garnered widespread attention in the field of oncology research in recent years. Its interaction with the tumor microenvironment (TME) and its prognostic value have become key research foci (17). The tumor microenvironment constitutes a complex ecosystem wherein platelets and lymphocytes play critical roles in tumor initiation, progression, and metastasis (18, 19). On one hand, platelets participate in the regulation of the microenvironment by releasing various bioactive factors. Studies demonstrate that pro-angiogenic factors secreted by platelets, such as vascular endothelial growth factor (VEGF) and platelet-derived growth factor (PDGF), effectively promote tumor angiogenesis. This provides essential nutritional support for tumor cell growth and metastasis (9, 20). On the other hand, lymphocytes, particularly cytotoxic T lymphocytes (CTLs) and natural killer (NK) cells, are central to the anti-tumor immune response, enabling the recognition and elimination of tumor cells (21). A low lymphocyte count typically indicates impaired anti-tumor immune function, creating favorable conditions for tumor cell proliferation and metastasis (22). Numerous clinical studies and meta-analyses have confirmed that an elevated PLR is significantly associated with reduced overall survival and an increased risk of tumor progression in patients with various malignancies. For instance, the research by Aoyama T et al. (23) identified PLR as a significant prognostic factor in gastric cancer. Furthermore, in breast cancer patients, higher PLR levels are significantly correlated with larger tumor size, lymph node metastasis, and decreased survival rates (24).

Mounting evidence in recent years has further established the significant prognostic value of the PLR in colorectal cancer (CRC) (25–28). The study by Bailón-Cuadrado M et al. (25) confirmed that PLR serves as an independent adverse prognostic factor for both overall survival and recurrence-free survival in patients undergoing curative resection for colon cancer. Large-scale meta-analyses have further strengthened this evidence, demonstrating that a high PLR is significantly associated with poor prognosis in CRC patients, with this correlation being particularly pronounced in advanced-stage patients (29).

However, traditional research primarily relies on single-time-point PLR measurements, which fail to capture the dynamic evolution of the inflammatory-immune microenvironment during treatment. Addressing this limitation, the present study innovatively applied a group-based trajectory modeling approach. By systematically collecting longitudinal PLR monitoring data at five distinct time points throughout the treatment course, we successfully identified three distinct trajectory patterns associated with significantly divergent prognoses. The survival analysis results showed that the 5-year overall survival rates of patients in the DG and IDG groups were significantly higher than those in the IG group (P < 0.05), indicating that a persistently declining PLR trajectory is closely associated with better long-term survival. Subgroup analysis revealed that in patients with stage III rectal cancer, the DG group had a superior 5-year OS compared with the IG group, whereas no significant difference was observed among the three groups in stage II patients. However, a formal interaction test using a Cox regression model did not detect a statistically significant interaction between TNM stage and trajectory groups (P = 0.374), suggesting that the observed subgroup difference might be attributable to limited sample size and insufficient statistical power. These findings warrant further validation in larger prospective cohorts.

In this study, there were significant differences in the distribution of N stage and TNM stage among different PLR trajectory groups, and this baseline imbalance might confound the survival analysis. To address this issue, we included T stage and N stage separately in the Cox regression models and constructed three stepwise−adjusted models. The results showed that in Model 2 and Model 3, the HRs for both the DG and IDG groups were statistically significant (P < 0.05), indicating that the prognostic value of PLR dynamic trajectories is independent of tumor stage. Nevertheless, caution is needed when interpreting the results, and future studies with larger sample sizes are required to further validate our findings using propensity score matching or prospective designs.

In addition, our univariate and multivariate analyses confirmed that age, N stage, and tumor differentiation grade are independent prognostic factors for patients with rectal cancer. Elderly patients often have more comorbidities and reduced physiological reserve, leading to poorer tolerance to surgery and adjuvant therapy, and consequently shorter overall survival (30). Tumor differentiation grade is also closely associated with prognosis; poorly differentiated tumors exhibit more aggressive biological behavior and are independent adverse prognostic factors (31). Together with the dynamic PLR trajectories, these three factors constitute a multidimensional prognostic evaluation system to inform clinical practice.

Previous studies (32, 33) have indicated that a high preoperative PLR is associated with an increased risk of infectious complications following surgery in patients with solid tumors, such as esophageal cancer and lung cancer. The underlying mechanism may relate to impaired lymphocyte-mediated immune surveillance. In this study, the main types of postoperative complications were pulmonary infection and anastomotic leakage. Notably, the incidence of Clavien-Dindo grade II or higher complications in the IG group was significantly higher than that in the DG group (P < 0.05). This finding suggests that a high PLR may contribute to the pathophysiological process of postoperative complications by impairing tissue repair capacity and increasing susceptibility to infection (23).

This study has several limitations. First, the single-center retrospective design and relatively small sample size may have resulted in insufficient statistical power for subgroup analyses and introduced a potential risk of selection bias. Second, the complication analysis in this study was confined to the 30-day postoperative period, whereas long-term complications (such as anastomotic stricture, chronic intestinal obstruction, and incisional hernia) could not be accurately assessed due to incomplete data documentation. Third, in this study, enrolled patients were required to have complete PLR data at time points T0 through T4 and to receive postoperative adjuvant chemotherapy, which excluded patients who experienced early postoperative death, severe complications, early recurrence, or intolerance to chemotherapy. This may have resulted in a study population biased toward a subgroup with better prognosis, potentially leading to an overestimation of the association between PLR trajectories and survival outcomes. Finally, there was a significant imbalance in N stage and TNM stage distribution among the PLR trajectory groups in this study. Although we adjusted for these factors using multivariable Cox regression models (with T stage and N stage entered separately), residual confounding may still exist. Due to the limited sample size, more sophisticated methods such as propensity score matching were not employed. Future studies should expand the sample size, adopt broader inclusion criteria, and employ prospective multicenter designs to enable a more comprehensive and in−depth evaluation of the role of PLR in the prognosis of rectal cancer.

## 5 Conclusion

In summary, this study innovatively applied GBTM to delineate the dynamic trajectories of PLR during the treatment period of patients with rectal cancer and found that these trajectories were associated with the risk of postoperative complications and long−term survival prognosis. Subgroup analysis suggested that PLR trajectories might have more pronounced prognostic value in patients with stage III rectal cancer; however, the interaction test did not reach statistical significance, and this finding requires further validation.

## Data Availability

The original contributions presented in the study are included in the article/**Supplementary Material**. Further inquiries can be directed to the corresponding author.
