# Supplementary material for: Association of dynamic platelet-to-lymphocyte ratio trajectories using group-based trajectory modeling with short-term outcomes and survival in patients undergoing laparoscopic radical resection for rectal cancer: a retrospective study
Source: Front Oncol. 2026 Jul 27;16:1819803. doi: 10.3389/fonc.2026.1819803 (PMC13453809; doi:10.3389/fonc.2026.1819803)
Supplement: Supplementary file 1 [file DataSheet1.docx]

**Supplement materials:**

Figure S1: ROC curve analysis of PLR for predicting 5-year survival in rectal cancer patients.

Figure S2: Kaplan-Meier survival curves by GBTM-derived subgroups in (A) stage II and (B) stage III rectal cancer patients.

Table S1: Model selection using Bayesian information criterion (BIC).

Table S2: Trajectory group characteristics derived from GBTM analysis.

Table S3: Characteristics of the three trajectory groups.

Table S4: Comparison of perioperative outcomes among groups.

Table S5: Univariable and multivariable Cox proportional hazards regression analysis examining factors associated with 5-years overall survival (Model 2).

**FIGURE S1**

ROC curve analysis of PLR for predicting 5-year survival in rectal cancer patients.

| **A**   | **B**   |
| --- | --- |
| **FIGURE S2**  Kaplan-Meier survival curves by GBTM-derived subgroups in **(A)** stage II and **(B)** stage III rectal cancer patients. | |

| **TABLE S1** Model selection using Bayesian information criterion (BIC). | | | | | | | | |  |
| --- | --- | --- | --- | --- | --- | --- | --- | --- | --- |
| Model | Groups | | Polynomial orders | | | BIC | | |  |
| 2-Group models | | | | | | | | |  |
| 1 | 2 | | 1 1 | | | -7150.42 | | |  |
| 2 | 2 | | 1 2 | | | -7148.97 | | |  |
| 3 | 2 | | 1 3 | | | -7149.47 | | |  |
| 4 | 2 | | 2 1 | | | -7118.49 | | |  |
| 5 | 2 | | 2 2 | | | -7116.9 | | |  |
| 6 | 2 | | 2 3 | | | -7117.26 | | |  |
| 7 | 2 | | 3 1 | | | -7119.65 | | |  |
| 8 | 2 | | 3 2 | | | -7118.05 | | |  |
| 9 | 2 | | 3 3 | | | -7118.4 | | |  |
| 3-Group models | | | | | | | | |  |
| 1 | 3 | | 1 1 1 | | | -6977.78 | | |  |
| 2 | 3 | | 1 1 2 | | | -6974.11 | | |  |
| 3 | 3 | | 1 1 3 | | | -6973.35 | | |  |
| 4 | 3 | | 1 2 1 | | | -6879.58 | | |  |
| 5 | 3 | | 1 2 2 | | | -6874.62 | | |  |
| 6 | 3 | | 1 2 3 | | | -6873.14 | | |  |
| 7 | 3 | | 1 3 1 | | | -6882.2 | | |  |
| 8 | 3 | | 1 3 2 | | | -6877.23 | | |  |
| 9 | 3 | | 1 3 3 | | | -6875.74 | | |  |
| 10 | 3 | | 2 1 1 | | | -6980.54 | | |  |
| 11 | 3 | | 2 1 2 | | | -6976.86 | | |  |
| 12 | 3 | | 2 1 3 | | | -6976.11 | | |  |
| 13 | 3 | | 2 2 1 | | | -6882.26 | | |  |
| 14 | 3 | | 2 2 2 | | | -6877.3 | | |  |
| 15 | 3 | | 2 2 3 | | | -6875.82 | | |  |
| 16 | 3 | | 2 3 1 | | | -6884.88 | | |  |
| 17 | 3 | | 2 3 2 | | | -6879.92 | | |  |
| 18 | 3 | | 2 3 3 | | | -6878.43 | | |  |
| 19 | 3 | | 3 1 1 | | | -6982.24 | | |  |
| 20 | 3 | | 3 1 2 | | | -6978.55 | | |  |
| 21 | 3 | | 3 1 3 | | | -6977.79 | | |  |
| 22 | 3 | | 3 2 1 | | | -6881.25 | | |  |
| 23 | 3 | | 3 2 2 | | | -6876.24 | | |  |
| 24 | 3 | | 3 2 3 | | | -6874.72 | | |  |
| 25 | 3 | | 3 3 1 | | | -6883.88 | | |  |
| 26 | 3 | | 3 3 2 | | | -6878.86 | | |  |
| 27 | 3 | | 3 3 3 | | | -6877.34 | | |  |
| BIC: Bayesian information criterion. | | | | | | | | |  |
| **TABLE S2** Trajectory group characteristics derived from GBTM analysis. | | | | | | | | | |
| Group | | Parameter | | Estimate | Std. Error | | T-value | P-value | |
| Increasing Group | | Intercept | | 315.66994 | 5.92738 | | 53.256 | 0.0000 | |
|  |  | **Linear** | | **-38.03729** | **1.74273** | | **-21.826** | **0.0000** | |
| Increasing-Decreasing Group | | Intercept | | 143.7057 | 12.35323 | | 11.633 | 0.0000 | |
|  |  | Linear | | 134.5628 | 9.37019 | | 14.361 | 0.0000 | |
|  |  | **Quadratic** | | **-23.14964** | **1.51357** | | **-15.295** | **0.0000** | |
| Decreasing Group | | Intercept | | 244.87613 | 13.59191 | | 18.016 | 0.0000 | |
|  |  | Linear | | 79.80085 | 10.31856 | | 7.734 | 0.0000 | |
|  |  | **Quadratic** | | **-6.62416** | **1.68747** | | **-3.925** | **0.0001** | |

| **TABLE S3** Characteristics of the three trajectory groups. | | | | |
| --- | --- | --- | --- | --- |
| Group | N | Proportion (%) | AvePP | OCC |
| Increasing Group | 67 | 27.2% | 0.986 | 188.2 |
| Increasing-Decreasing Group | 90 | 36.6% | 0.995 | 344.9 |
| Decreasing Group | 89 | 36.2% | 0.982 | 96.2 |
| AvePP：Average posterior probability. OCC: Odds of correct classification.  Total N = 246. | | | | |

| **TABLE S4** Comparison of perioperative outcomes among groups. | | | | | |
| --- | --- | --- | --- | --- | --- |
| Characteristic | IG (N=67) | IDG(N=90) | DG (N=89) | F/χ² | *P* value |
| Intraoperative blood loss (ml) | 46.67 ± 11.60 | 48.01 ± 10.82 | 46.00 ± 10.68 | 0.77 | 0.463 |
| Operative time (min) | 177.40 ± 21.06 | 172.71 ± 21.35 | 179.78 ± 22.78 | 2.42 | 0.091 |
| Postoperative hospital stay (days) | 9.78 ± 2.58 | 9.40 ± 2.63 | 9.36 ± 2.09 | 0.65 | 0.522 |
| Total retrieved lymph nodes | 24.94 ± 4.99 | 23.67 ± 5.39 | 24.44 ± 4.24 | 1.36 | 0.258 |
| CD grade |  |  |  |  |  |
| GradeⅠ | 53 (79.1%) | 80 (88.9%) | 82 (92.1%) | 6.18 | 0.046 |
| ≥Grade Ⅱ | 14 (20.9%) | 10 (11.1%) | 7 (7.9%) |  |  |
| CD grade: Clavien-Dindo classification of surgical complications. | | | | | |

| **TABLE S5** Univariable and multivariable Cox proportional hazards regression analysis examining factors associated with 5-years overall survival (Model 2). | | | | |
| --- | --- | --- | --- | --- |
| **Variable** | **Univariable** | ***P* value** | **Multivariable** | ***P* value** |
|  | **HR (95% CI)** |  | **HR (95% CI)** |  |
| Sex |  |  |  |  |
| Male | Ref. |  |  |  |
| Female | 1.071 (0.686 - 1.671) | 0.764 |  |  |
| Age, years |  |  |  |  |
| ＜65 | Ref. |  | Ref. |  |
| ≥65 | 1.908 (1.191 - 3.056) | 0.007 | 1.803 (1.110 - 2.929) | 0.017 |
| ASA Class |  |  |  |  |
| Ⅰ | Ref. |  |  |  |
| Ⅱ | 0.972 (0.599 - 1.579) | 0.910 |  |  |
| Ⅲ | 0.718 (0.338 - 1.526) | 0.389 |  |  |
| BMI |  |  |  |  |
| ＜23.5 | Ref. |  |  |  |
| ≥23.5 | 1.315 (0.831 - 2.081) | 0.242 |  |  |
| Hypertension |  |  |  |  |
| No | Ref. |  |  |  |
| Yes | 0.961 (0.615 - 1.501) | 0.861 |  |  |
| Diabetes |  |  |  |  |
| No | Ref. |  |  |  |
| Yes | 1.469 (0.858 - 2.516) | 0.161 |  |  |
| Surgical Approach |  |  |  |  |
| Open | Ref. |  |  |  |
| Laparoscopic | 1.591 (0.221 - 11.441) | 0.645 |  |  |
| Tumor Size |  |  |  |  |
| ＜3.5 | Ref. |  |  |  |
| ≥3.5 | 1.202 (0.762 - 1.897) | 0.429 |  |  |
| CEA | 1.080 (0.899–1.300) | 0.412 |  |  |
| CA199 | 1.010 (0.983–1.030) | 0.570 |  |  |
| T Stage |  |  |  |  |
| T_1_-T_2_ | Ref. |  | Ref. |  |
| T_3_-T_4_ | 1.677 (0.412–6.829) | 0.470 | 1.620 (0.355 - 7.382) | 0.533 |
| N Stage |  |  |  |  |
| N_0_ | Ref. |  | Ref. |  |
| N_1_ | 1.505 (0.817–2.772) | 0.189 | 1.690 (0.876 - 3.262) | 0.117 |
| N_2_ | 2.363 (1.440–3.876) | <0.001 | 1.724 (1.023 - 2.902) | 0.041 |
| TNM Stage |  |  |  |  |
| Ⅱ | Ref. |  |  |  |
| Ⅲ | 1.988 (1.268 - 3.118) | 0.003 |  |  |
| Perineural Invasion |  |  |  |  |
| No | Ref. |  | Ref. |  |
| Yes | 1.603 (1.012 - 2.537) | 0.044 | 1.323 (0.826 - 2.118) | 0.244 |
| Vascular Invasion |  |  |  |  |
| No | Ref. |  | Ref. |  |
| Yes | 1.994 (1.238 - 3.210) | 0.005 | 1.445 (0.868 - 2.405) | 0.157 |
| Differentiation |  |  |  |  |
| Well-Moderate | Ref. |  | Ref. |  |
| Poor | 2.066 (1.312 - 3.253) | 0.002 | 2.009 (1.259 - 3.207) | 0.003 |
| CD grade |  |  |  |  |
| GradeⅠ | Ref. |  |  |  |
| ≥Grade Ⅱ | 1.299 (0.702–2.404) | 0.404 |  |  |
| Chemotherapy completion |  |  |  |  |
| No | Ref. |  |  |  |
| Yes | 0.604 (0.344–1.061) | 0.080 | 0.700 (0.393 – 1.247) | 0.226 |
| Trajectory Group |  |  |  |  |
| IG | Ref. |  | Ref. |  |
| IDG | 0.517 (0.307 - 0.871) | 0.013 | 0.530 (0.310 - 0.907) | 0.020 |
| DG | 0.391 (0.225 - 0.681) | < 0.001 | 0.394 (0.219 - 0.709) | 0.002 |
